# Postmortem fluorescence angiography of the explanted human heart

**DOI:** 10.1007/s00414-021-02730-9

**Published:** 2021-11-28

**Authors:** Constantin Lux, Miriam Klinger, Patrick Sauer, Marcel A. Verhoff, Mattias Kettner

**Affiliations:** Institute of Legal Medicine, Goethe University, Kennedyallee 104, 60596 Frankfurt am Main, Germany

**Keywords:** Postmortem, Fluorescence, Angiography, Indocyanine green, ICG, Heart

## Abstract

Within the scope of this technical report, the feasibility of indocyanine green (ICG) as a fluorescent agent for postmortem angiography of the heart is tested. The study included 4 deceased persons with no respective medical history of heart diseases. The basic patterns of findings in ICG fluorescence angiography associated with healthy hearts are presented. The method can easily be integrated into a workflow without restricting the macroscopic or histologic diagnostics. This paper represents the fundamental technical and analytical basis for upcoming studies concerning the possibilities and limitations of fluorescence angiography in the diagnosis of heart pathology.

## Introduction

The basis of postmortem examination of cardiac pathology is represented by the combination of macroscopic and microscopic evaluation. Using contrast agents to visualize the course of coronary arteries might give insights into pathologic mechanisms concerning coronary blood flow; however, the chosen method must not impair macroscopic or histologic evaluation. Postmortem visualization of the vascular system of the heart has been subject to medical research in the past [1–4]. In 1928, Campbell examined coronary circulation using barium injections [5]. Newer studies primarily focus on the use of postmortem computed tomography in combination with contrast agents [6, 7]. Recently, thermography has been presented as a feasible method preserving the myocardial structure [8].

Since its approval in 1959 (FDA NDA 011,535), indocyanine green (ICG) has been widely used in many medical disciplines as a fluorescent contrast agent for the visualization of structures and functions of the body [9–13]. Clinical applications include intraoperative visualization of lymphatic structures of the blood vessels in tumor surgery [9, 11], documentation of leaking retinal blood vessels [10], and intraoperative evaluation of coronary bypass perfusion in cardiosurgery [13]. Based on clinical application results, a postmortem use appears to be promising and feasible, e.g., to demonstrate perfusion deficits of internal organs with a rather minor technical effort.

For most clinical applications, 5 mg of ICG is injected into the venous system. As the cardiovascular or lymphatic system provides circulation and thus the distribution of the substance (ICG) in the living body and times from injection until the beginning of arterial/venous phases correspond to usual clinical applications using contrast agents, no comparable protocol usable for a postmortem application exists in the clinical setting. Since clinical intraoperative applications are usually based on the use of expensive technology such as operation robots [9], which precludes the technique from easy access to the forensic setting, an easy to obtain and inexpensive protocol and setup is presented in this study. Since heart disease and associated pathological conditions of the coronary arteries are frequent and relevant findings in autopsies and the anatomy of the heart and its coronary arteries are easily accessible, the heart was chosen as the exemplary organ to examine the feasibility of postmortem ICG application. Angiographical and physiological aspects and findings of examinations of hearts without relevant cardiac pathologies are presented.

## Material and methods

The organ specimen originated from autopsies carried out at the Frankfurt Institute of Legal Medicine. The study was approved by the ethical committee of the Faculty of Medicine, Goethe-University of Frankfurt/Main. Since the autopsies were carried out as medicolegal autopsies, permission was obtained from the prosecutor’s office. The choice of organs suitable for inclusion in the study was based on the absence of clinically known pre-existing medical conditions (as stated in medical reports and obituaries) as well as autopsy findings up to the point of heart removal. In this study, 4 cases with macroscopically inconspicuous hearts and no respective medical history were examined (Table 1).

**Table 1:** Cases included in the study; *M*, male; *F*, female

Case no	Age	Gender	Heart mass	Cause of death
1	12	M	195 g	Hanging
2	46	F	330 g	Intoxication
3	35	F	389 g	Intoxication
4	47	F	320 g	Gastrointestinal bleeding

### Preparatory and perfusion procedures

The hearts were extracted at the pericardial boundaries. Epicardial fat tissue surrounding the coronary ostia was removed. Button cannulas were inserted into the coronary boundaries and externally fixated with a ligature. In order to compensate for the gravity-related pressure distribution within the capillary system (in a hanging position), the organs were suspended in an isotonic saline solution. Using a Dodge embalming machine (Dodge 709,406/V2, The Mazwell Group, Hampshire, UK), 100 μg ICG contrast agent (Diagnostic Green GmbH, Aschheim, Germany) dissolved in 500 ml H_2_O (0.9% sodium chloride) was perfused for 3 min per coronary artery at a pressure of 0.1 bar.

### Fluorescence imaging and documentation (Fig. *1*)

**Fig. 1:**
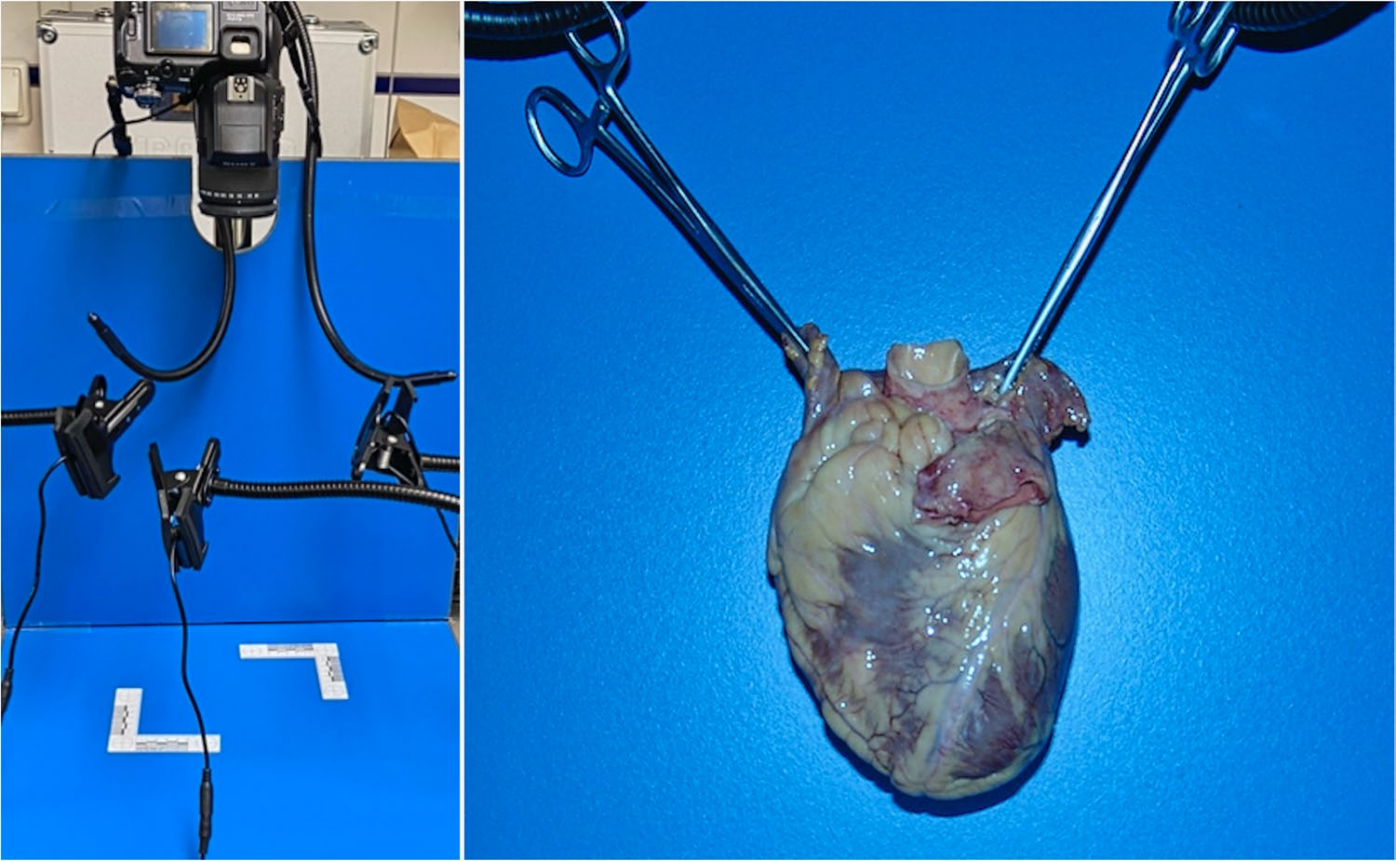
*Left:* Setup prepared for the documentation of ICG-perfused heart with three laser diodes, camera, and filters. *Right*: Illustrative documentation of the ventral surface of a heart after perfusion

To document perfusion, the exterior surfaces of the heart, the endocardium, and standardized regions of the myocardium (anterior wall, posterior wall, septum, and flat sections thereof) were irradiated with 3 laser diodes (RLDB780-30–3, 785 nm, 30 mW, Roithner Lasertechnik, Vienna, Austria). The total exposure intensity on an area of 30 × 30 cm was computed to be 0.1 mW/cm^2^ cumulatively. The sensor was a modified bridge camera (Sony DSC f828, 8 megapixels, Sony, Minato, Tokyo, Japan) with a magnetically retractable lock filter so that manual settings could be chosen for the relevant parameters (exposure time: 25 s; aperture: 8; ISO: 64). A coated long-pass filter (FELH800, Thorlabs, Newton, NJ) was used to filter out the excitating light from the laser diodes. The pictures were taken in a darkened room after 3 min of perfusion. A total of 14 standardized exposures were made per organ. These included normal digital recordings, as well as recordings with the described filter system from each side of the heart (ventral, dorsobasal, right, left), a recording of the endocardium of both opened ventricles, and an overview of the flat sections of the anterior and posterior walls and the septum. Digital photo files were analyzed in raw image format using the software program GraphicConverter 11 (Lemke Software GmbH, Peine, Germany). Using the abovementioned manual camera settings and standard ICG solution, no digital image editing was necessary.

### Histological examinations

Histological examinations were carried out on standardized samples (anterior and posterior wall, septum, right heart chamber myocardium, left and right atrium, AV nodes, and the His bundle) as well as macroscopically and/or angiographically conspicuous cardiac muscle tissue. Hematoxylin–eosin (HE) and Azan stainings were employed as the standard examination routine. The microscopical examination was carried out at a primary magnification of up to 400 × . No artifacts or restrictions concerning the histological examination and diagnosis due to ICG perfusion were observed.

## Results

**Fig. 2:**
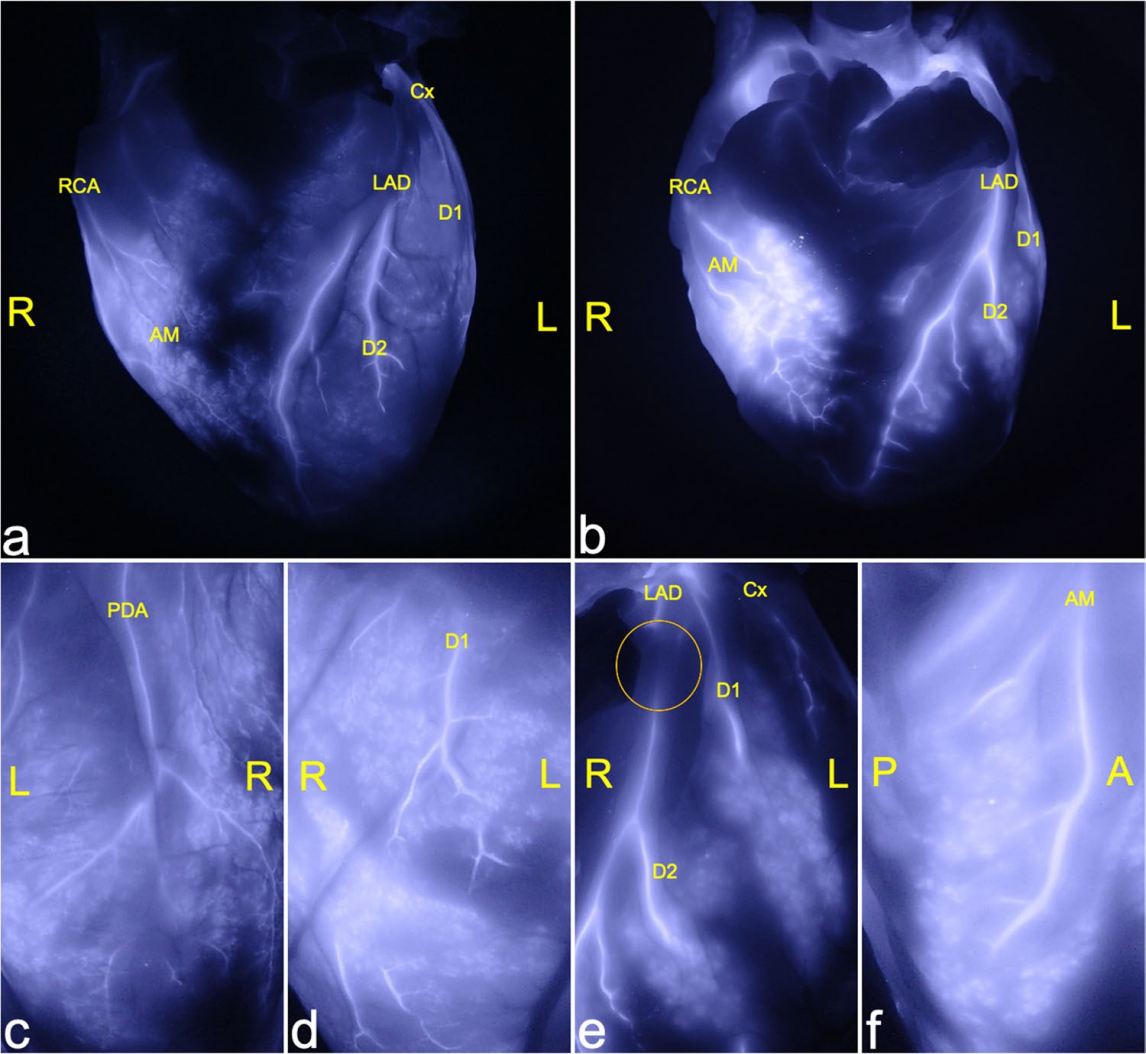
*Regular fluorescence image of the heart.* **a**, **b** Ventral overviews of the heart of a 12-year-old child (**a**; case 1) and a 46-year-old woman (**b**: case 2) without pathologic findings and a typical, deciduous tree-like branching of the coronary arteries; **c**–**d** magnification of posterior descending artery (**c**, case 1) and diagonal branches of the left anterior descending artery (**d**; case 1) with the typical leave-tree-like branching and presentation of end arterial and capillary vessels; diagonal branches of the left anterior descending artery with fluorescence signal interruption (circle) due to a muscle bridge (**e**; case 2) and the right marginal artery (**f**; case 2) without pathologic findings. LAD, left anterior descending artery; Cx, circumflex artery; D1, D2, diagonal branches; RCA, right coronary artery; AM, (right) acute marginal branch; PDA, posterior descending artery; L, left; R, right; P, posterior; A, anterior

Fluorescence analysis after ICG application showed detailed visualization of the three main branches (LAD, LCX, and RCA) of the coronary artery system and their respective arterioles and capillary trees. None of the included cases with macroscopically inconspicuous hearts displayed any pathological findings, e.g., caliber variations along with their courses, kinking of vessels, or endothelial wall pathologies. In all cases, angiographic examination revealed a continuous reduction of the diameter concordant with regular branching. Cardiac muscle tissue showed a deciduous tree-like fluorescence in the vicinity of intensively fluorescent vessels (Fig. 2). Fluorescence was seen best and most uniformly distributed after 3 min of perfusion.

## Discussion

Coronary heart disease, myocarditis, endocarditis, and cardiomyopathies belong to the most common pathological conditions of the heart [14]. Many of the pathophysiological processes that are relevant to the occurrence of death are associated with changes in the endarterial and capillary zones of the myocardium. In this study, it was shown that postmortem fluorescence angiography of the heart using ICG to delineate vascular courses down to the capillary tree is usable. Here, digital overviews of the heart (before meticulous preparation) and assessment of the subendothelial vessel courses in conjunction with fluorescence angiography of the myocardium on plane sections proved to be a useful method to supplement routine macroscopic and microscopic examinations.

In fluorescence angiography, the so-called optical or diagnostic window, which enables particularly deep optical penetration into tissue without an ionizing effect of the radiation, is of particular interest. The majority of information available is based on the range of 1300- to 650-nm wavelengths. The suitability of ICG for intraoperative vascular imaging is primarily based on its absorption and emission spectrum in the near-infrared range (wavelengths between 780 nm and 3 μm), which enables deep tissue penetration and allows for optimal assessment of vessel courses through fat and connective tissue layers. Discontinuation of fluorescence may indicate a disorder of tissue perfusion, i.e. may serve as an indicator of occlusion, while focal hyperfluorescence may indicate increased permeability of vessel walls due to disease or trauma.

Understanding possible artifacts and their causes is essential for the correct understanding of the findings. Perfusion artifacts may be created through a vascular outlet ligature, e.g., ligature of the LCX during the fixation process of button cannulas. Technical artifacts can occur in the form of inhomogeneous illumination, reflections, overexposure, and incorrect contrast agent concentrations. These should be considered in particular if no macroscopic and/or micromorphological correlations are found for the fluorescence angiographic findings. In this study, no detrimental artifacts or other alterations impeding histological assessment were noticed.

## Conclusions and outlook

Fluorescence angiography of the heart might be suitable to document pathological changes of the blood supply of the heart and may serve to complement routine macroscopic as well as microscopic examination. Further studies will focus on the question, to what extent fluorescence angiography findings may help to narrow the diagnostic gap between macroscopical and microscopical findings in pathologies associated with coronary heart disease and inflammatory diseases of the heart.
